# Voxel-based morphometry reveals the correlation between gray matter volume and serum P-tau-181 in type 2 diabetes mellitus patients with different HbA1c levels

**DOI:** 10.3389/fnins.2023.1202374

**Published:** 2023-05-15

**Authors:** Yian Gao, Chaofan Sui, Boyao Chen, Haotian Xin, Yena Che, Xinyue Zhang, Na Wang, Yuanyuan Wang, Changhu Liang

**Affiliations:** ^1^Department of Radiology, Shandong Provincial Hospital Affiliated to Shandong First Medical University, Jinan, Shandong, China; ^2^College of Radiology, Shandong First Medical University (Shandong Academy of Medical Sciences), Tai’an, Shandong, China; ^3^Department of Radiology, Shandong Provincial Hospital, Cheeloo College of Medicine, Shandong University, Jinan, Shandong, China; ^4^Department of Medical Imaging, Binzhou Medical University, Yantai, Shandong, China

**Keywords:** glycated hemoglobin, type 2 diabetes mellitus, gray matter volume, tau phosphorylated at threonine 181, voxel-based morphometry

## Abstract

**Introduction:**

Emerging evidence suggested widespread decreased gray matter volume (GMV) and tau hyperphosphorylation were associated with type 2 diabetes mellitus (T2DM). Insulin resistance is one of the mechanisms of neuron degeneration in T2DM; it can decrease the activity of protein kinase B and increase the activity of glycogen synthesis kinase-3β, thus promoting the hyperphosphorylation of tau protein and finally leading to neuronal degeneration. However, the association between GMV and serum tau protein phosphorylated at threonine 181 (P-tau-181) in T2DM patients lacks neuroimaging evidence. We aimed to investigate the difference in brain GMV between T2DM patients with different glycated hemoglobin A1c (HbA1c) levels and healthy control (HC) subjects and the correlation between serum P-tau-181 and GMV in T2DM patients.

**Methods:**

Clinical parameters, biochemical indicators, and MRI data were collected for 41 T2DM patients with high glycosylated hemoglobin level (HGL), 17 T2DM patients with normal glycosylated hemoglobin level (NGL), and 42 HC subjects. Voxel-based morphometry (VBM) method was applied to investigate GMV differences among groups, and multiple regression analysis was used to examine the correlation between serum P-tau-181 and GMV.

**Results:**

Compared with HC subjects, the T2DM patients with HGL or NGL all showed significantly decreased GMV. Briefly, the GMV decreased in T2DM patients with HGL was mainly in the bilateral parahippocampal gyrus (PHG), right middle temporal gyrus (MTG), temporal pole (TPOmid), hippocampus (HIP), and left lingual gyrus. The GMV reduction in T2DM patients with NGL was in the right superior temporal gyrus (STG), and there was no significant difference in GMV between the two diabetic groups. The GMV values of bilateral PHG, right MTG, TPOmid, HIP, and STG can significantly (*p* < 0.0001) distinguish T2DM patients from HC subjects in ROC curve analysis. In addition, we found that serum P-tau-181 levels were positively correlated with GMV in the right superior and middle occipital gyrus and cuneus, and negatively correlated with GMV in the right inferior temporal gyrus in T2DM patients.

**Conclusion:**

Our study shows that GMV atrophy can be used as a potential biological indicator of T2DM and also emphasizes the important role of P-tau-181 in diabetic brain injury, providing new insights into the neuropathological mechanism of diabetic encephalopathy.

## 1. Introduction

Diabetes mellitus (DM) is a metabolic disease characterized by chronic hyperglycemia caused by insufficient insulin secretion or defective insulin action (Classification and Diagnosis of Diabetes, 2022). Long-term complications of DM include progressive end-organ damage to the central nervous system, cardiovascular system, kidneys, eyes, and peripheral nervous system (Chen et al., 2012; Yazdanpanah et al., 2017; Yao et al., 2021). DM directly affects structural and metabolic alterations in the brain according to earlier research (Seaquist, 2010; García-Casares et al., 2014; Seaquist, 2015). Patients with type 2 diabetes mellitus (T2DM) had less gray matter volume (GMV) in the corticostriatal marginal area according to the structural MRI studies (Brundel et al., 2010; García-Casares et al., 2014). Research has demonstrated that decreased GMV in T2DM patients will result in default mode network dysfunction, which affects working memory and emotional processing and is linked to abnormalities in cognition, anxiety, and depressive regulation (Chen et al., 2016; Yao et al., 2021). It has been confirmed that cognitive function is positively correlated with GMV in patients with T2DM, and the decrease of GMV may lead to cognitive dysfunction (Roy et al., 2020; Yao et al., 2021). Clinical manifestations associated with diabetic brain damage include decreased cognitive, executive, and memory abilities (Brundel et al., 2012; Groeneveld et al., 2018; Hoyos et al., 2022), which increase the risk of vascular dementia and Alzheimer’s disease (AD) in patients with T2DM (De la Monte and Tong, 2009; García-Casares et al., 2014; Hoyos et al., 2022). In addition, metabolic disturbances associated with T2DM affect a variety of biochemical pathways, which may also be responsible for neuronal dysfunction and cognitive decline (Yaffe et al., 2011; García-Casares et al., 2014).

The widely used indicator of glycemic management is glycosylated hemoglobin A1c (HbA1c), and it has been recognized as an acceptable indicator of long-term glycemic control by the Federal Drug Association (FDA), American Diabetes Association (ADA), and Canadian Diabetes Association (CDA) (American Diabetes Association, 2011; Yazdanpanah et al., 2017). High HbA1c levels have been linked to harm to brain structure, especially the hippocampus, according to earlier research (Geijselaers et al., 2015; Garfield et al., 2021). Additionally, an increasing number of studies have found that DM damages the integrity of the blood–brain barrier (BBB) structure, increasing its permeability, and causes cerebral hemodynamic impairment. Additionally, it has been demonstrated that tau pathology can cause BBB harm, whereas BBB dysfunction can result in tau hyperphosphorylation (Bogush et al., 2017; Qiao et al., 2020; Oliveira et al., 2021). The BBB is a highly selective semipermeable membrane that separates blood from nerve tissue. Under physiologically normal circumstances, the blood–brain barrier tightly regulates the chemicals that enter and leave the brain tissue, maintains the microenvironment homeostasis of the central nervous system, and guards against immune cells and toxins damaging brain cells (Coucha et al., 2018). Additionally, the blood–brain barrier has several specialized transporters, such as P-glycoprotein (gp), which may remove harmful amyloid β (Aβ) from brain tissue (Deo et al., 2014). The hyperphosphorylation of tau protein leads to the formation of neurofibrillary tangles (NFTs), resulting in the impairment of normal axonal transport, synaptic loss, and neuronal function impairment, which drives neurodegeneration and is closely related to T2DM (Ke et al., 2009; Oliveira et al., 2021). Smaller brain volumes in individuals with DM and white matter lesions have also been observed in neuroimaging studies, and these findings are likely to be connected to BBB degradation (Bogush et al., 2017). We thus proposed the following hypothesis: reduced gray matter volume in diabetes patients results in improper blood–brain barrier function, which, in turn, results in abnormal P-tau-181 metabolism.

At present, due to the lack of reliable and sensitive biomarkers, the definite diagnosis of diabetic brain injury is mainly based on psychological and clinical manifestations. The ability to research diabetic brain changes using brain imaging and image analytic techniques gave us the chance to examine the precise quantitative topological features of the brain structure. With its total automation, standardization, high accuracy, and dependability, voxel-based morphometry (VBM) is a traditional automatic whole-brain analysis approach that allows unbiased research to be performed regarding variations in gray matter between patients and controls (Ashburner and Friston, 2000). The study of the diabetic brain structure has made extensive use of and acceptance of the VBM approach (Chen et al., 2012; García-Casares et al., 2014; Liu et al., 2017; Wu et al., 2017; He et al., 2022). According to recent research, patients with AD dementia have higher serum P-tau-181 levels (Mielke et al., 2018), which correlate with the levels of tau protein in the cerebrospinal fluid and can also predict the progression of the disease in participants with normal cognitive function and in patients with mild cognitive impairment (Janelidze et al., 2020; Thijssen et al., 2020; Karikari et al., 2021). Moreover, a study has shown that plasma p-tau181 can be used in clinical settings as a blood-based predictive biomarker for cognitive deterioration (Clark et al., 2021). However, no studies have investigated the changes in GMV in T2DM patients with different HbA1c levels and the relationship between the changes in GMV and the metabolism of serum P-Tau-181 protein in T2DM patients.

Therefore, in this study, we classified patients with T2DM into two groups according to their HbA1c levels and included healthy controls (Qaseem et al., 2018; Classification and Diagnosis of Diabetes, 2020). We aimed to compare the significant difference in GMV among the three groups and to analyze the correlation between serum P-tau-181 levels and GMV in T2DM patients, thereby providing a diagnostic biomarker of diabetic brain injury.

## 2. Materials and methods

### 2.1. Subjects

The Shandong Provincial Hospital Affiliated to Shandong First Medical University Subcommittee on Human Studies Institutional Review Board examined and approved the study. Before the study began, informed consent was acquired from each participant. All patients met the 1999 World Health Organization T2DM diagnostic criteria. Inclusion criteria for all patients are presented in the Supplementary materials. Current diabetes mellitus management recommendations from the American Diabetes Association (ADA) call for a baseline HbA1c goal of 7.0% for nonpregnant persons with diabetes (Qaseem et al., 2018; Classification and Diagnosis of Diabetes, 2020); therefore, we defined high HbA1c level (HGL) as the HbA1c level above 7.0%. Forty-one T2DM patients with HGL (age: 57.44 ± 9.4 years; 20 females and 21 males) and 17 T2DM patients with normal HbA1c level (NGL) (age: 59.71 ± 9.674 years; 7 females and 10 males) were recruited from outpatient clinics in Shandong Provincial Hospital between January 2022 and December 2022, and 42 healthy control (HC) subjects (age: 54.19 ± 8.194 years; 23 females and 19 males) were also included in our study.

The inclusion criteria for all patients were as follows: (1) an age range from 40 to 70 years; (2) junior high school education or higher; and (3) right-handedness. Patients were excluded from the study if they had (1) left-handedness; (2) neuropathies caused by other reasons; (3) brain trauma, surgery, or brain tumors; (4) acute complications of T2DM and severe hypertension; (5) history of any serious cerebrovascular, neurological, or psychiatric diseases; (6) abuse of alcoholism or drugs; or (7) any contraindications to MRI. The inclusion criteria for the HCs were as follows: (1) age 40–70 years, with junior high school education or above; (2) right-handed; (3) no history of diabetes and glycated hemoglobin (HbA1c) level of between 4 and 6%; (4) no history of any serious medical, psychiatric or neurologic diseases; (5) no history of head trauma, surgery, tumors or loss of consciousness; and (6) absence of alcohol or drug abuse.

### 2.2. Clinical assessment

All study subjects were evaluated with the Montreal Cognitive Assessment (MoCA) Beijing version^1^ by the measurers designated by the research group. To ensure the homogeneity of measurement results, all measurers were trained in the same institution, and the expert group members conducted homogeneous evaluation on the measurers to ensure the homogeneity of measurement results. Plasma samples were obtained from all subjects by venous puncture, and a total of 5 mL of venous blood was drawn with an EDTA tube for anticoagulation and centrifuged at 3000 r/min for 15 min; the separated plasma was stored at −20°C and concentrated at −70°C for detection. Enzyme-linked immunosorbent assay (ELISA) was performed according to the instructions of the Tau (Phospho) [pT 181] Human ELISA Kit provided by Thermo Fisher/Invitrogen, United States. All specimens were tested with the same lot of reagents.

### 2.3. Image acquisition

All participants were imaged utilizing a 32-channel head coil product on a MAGNETOM Skyra 3.0 T MR scanner (Siemens Healthcare, Erlangen, Germany). The three-dimensional T1-weighted (3DT1W) rapid acquisition gradient-echo sequence with the following settings was used to capture the structural images: TR/TE = 2300/2.32 ms, slice thickness = 0.9 mm, the number of slices = 213, and FOV = 24 × 24 cm^2^. To further identify any anomalies in the brain, T2W fluid-attenuated inversion recovery and T2W turbo spin echo were acquired. All participants held motionless during the scan.

### 2.4. Data preprocessing

Following data acquisition, 3D T1W image processing was carried out using VBM with DARTEL (Diffeomorphic Anatomical Registration Through Exponentiated Lie Algebra) (Ashburner, 2007) method based on the statistical parametric mapping (SPM8,^2^) toolbox. Throughout the iterative unified model, DARTEL’s fully deformable registration and normalization method ensures precise intersubject alignment. The anterior commissure (AC), posterior commissure (PC), and mid-sagittal plane were used to manually identify landmarks to match all of the 3D T1W pictures to conventional AC-PC space. The New Segment tool in SPM was then used to segment the aligned images into gray matter (GM), white matter (WM), and cerebrospinal fluid (CSF) in native space with unified segmentation. The next step was to tightly modify each of the segmented GM images to create a set of aligned GM images. The DARTEL algorithm was then applied to the serial images from all the individuals who had been aligned to build the study-specific GM templates. All aligned GM images were distorted to the template during template generation, producing a number of flow fields that parameterized the deformation. After spatial normalization to Montreal Neurological Institute (MNI) space, the GM images were modulated to correct local volume expansion due to the nonlinear component of the spatial transformation. The generated gray matter volume (GMV) images were then smoothed with an isotropic Gaussian kernel of 8 mm full width at half maximum (FWHM).

### 2.5. Statistical analysis

Statistical analysis of demographic data and clinical parameters was performed using SPSS Version 24.0 (SPSS Inc., Chicago, IL, United States). Shapiro–Wilk test(S-W test) was used for the normality test, then we used one-way ANOVA to analyze the clinical parameters of the three groups. Nonparametric tests were used for data that did not meet the normality test and sex differences were tested using the Chi-square test (*χ*^2^).

To analyze the differences in GMV images among the three groups, voxel-wise one-way analysis of covariance (ANCOVA) was performed using SPM 8, with age and gender as covariates (we readded education level as covariates in Supplementary materials, the results are shown in Supplementary Table S1), and the analysis was constrained in the binary AAL template as mask (Tzourio-Mazoyer et al., 2002). The significant results were derived after using the cluster-level false discovery rate (FDR) multiple comparisons correction (cluster-wise threshold of *q* < 0.05 based on an uncorrected voxel-wise threshold of *p* < 0.001). For each significantly altered cluster identified by ANCOVA, two-sample t-tests with cluster-level FDR correction (voxel-wise *p* < 0.001, cluster-wise *q* < 0.05) were applied within that cluster to compare differences between two groups. The anatomical positions of clusters were identified using Xjview software^3^ and finally visualized by Mricron software.

The voxel-wise correlation between GMV and serum P-tau-181 for all T2DM patients was analyzed using multiple regression in SPM 8, while controlling age and gender as covariates and using the binary AAL template as mask. Multiple comparisons were also corrected using the cluster-level FDR method (voxel-wise *p* < 0.001, cluster-wise *q* < 0.05). In addition, we used both P-tau-181 and HbA1c as independent variables in multiple regression to investigate their combined effects on GMV (the details are presented in the Supplementary materials).

For significantly different clusters between groups, we extracted the mean values within clusters for receiver operating characteristic (ROC) curves to assess each value’s potential to serve as a crucial neuroimaging biomarker to distinguish between T2DM with HGL group, T2DM with NGL group, and HC group. The area under the curve (AUC) was then calculated using the MedCalc Statistical Software.^4^ We calculated the corresponding optimal cutoff point, the maximum Youden index (Fluss et al., 2005), specificity, sensitivity, and 95% confidence intervals (CIs). Finally, the DeLong test (DeLong et al., 1988) was used to statistically compare the AUC for each mean value.

## 3. Results

### 3.1. Demographic and clinical characteristics

The demographic information and clinical data of the three groups are displayed in Table 1. Age, education attainment, or MoCA scores did not significantly differ among the three groups. Additionally, no notable variations were discovered in duration of disease, GMV, P-tau-181, total cholesterol, triglycerides, high-density lipoprotein (HDL), or low-density lipoprotein (LDL) between the T2DM with HGL group and T2DM with NGL group; however, the T2DM with HGL group had significantly higher fasting blood glucose (Glu) and HbA1c.

**Table 1:** Demographic and cognitive characteristics of T2DM patients and healthy controls.

Characteristic	HGL	NGL	HC	*p* value (ANOVA/*χ*^2^)	*P* value (post-hoc)		
					HGL vs. HC	HGL vs. NGL	NGL vs. HC
Gender	21 M/20 F	10 M/7 F	19 M/23 F	0.627^*χ*2^	-	-	-
Age (y)	57.44 ± 9.4	59.71 ± 9.674	54.19 ± 8.194	0.072^a^	-	-	-
Education (y)	12.29 ± 3.64	11.82 ± 3.23	13.90 ± 4.11	0.073^a^	-	-	-
MoCA (score)	24.00 ± 3.33	25.41 ± 2.78	25.28 ± 3.37	0.142^a^	-	-	-
Duration (y)	11.83 ± 8.41	7.82 ± 8.06	-	0.1^t^	-	-	-
Smoke, n (%)	11(27)	4(23)	4(9)	0.116^*χ*2^	-	-	-
Hypertension, n (%)	23(56)	7(41)	8(19)	0.002^*χ*2^	<0.001	N.S	N.S
Glu (mmol/l)	8.9(7.85 ~ 11.21)	6.86(6.36 ~ 7.75)	5.49(4.74 ~ 6.09)	<0.001	<0.001	0.022	0.006
HbA1c (%)	9.40 ± 1.72	6.63 ± 0.22	5.77 ± 0.35	<0.001^a^	<0.001	<0.001	0.009
BMI (kg/m^2^)	26.21 ± 2.83	25.30 ± 2.93	23.93 ± 3.35	0.004	0.001	0.311	0.124
CHOL (mmol/l)	5.02 ± 1.20	4.76 ± 1.06	5.08 ± 1.04	0.554^a^	-	-	-
TG (mmol/l)	1.80 ± 1.01	1.72 ± 1.23	1.25 ± 0.54	0.017^a^	0.006	0.766	0.069
HDL (mmol/l)	1.27 ± 0.40	1.36 ± 0.23	1.52 ± 0.34	0.007^a^	0.002	0.399	0.115
LDL (mmol/l)	3.1 ± 0.91	2.73 ± 0.88	3.02 ± 0.81	0.25^a^	-	-	-
GMV (mm^3^)	528.86 ± 42.46	541.61 ± 26.27	588.00 ± 45.49	<0.001^a^	<0.001	0.290	<0.001
P-Tau-181	312.02 ± 56.07	301.42 ± 64.53	276.86 ± 56.56	0.022^a^	0.007	0.544	0.160

HGL, T2DM patients with HGL; NGL, T2DM patients with NGL; HC, healthy controls; GMV, gray matter volume; *χ*^2^, Chi-square test; a, ANOVA test; MoCA, Montreal Cognitive Assessment; *t*, two-sample *t* test; N.S, not significant.

### 3.2. Altered gray matter volume among groups

**Figure 1:**
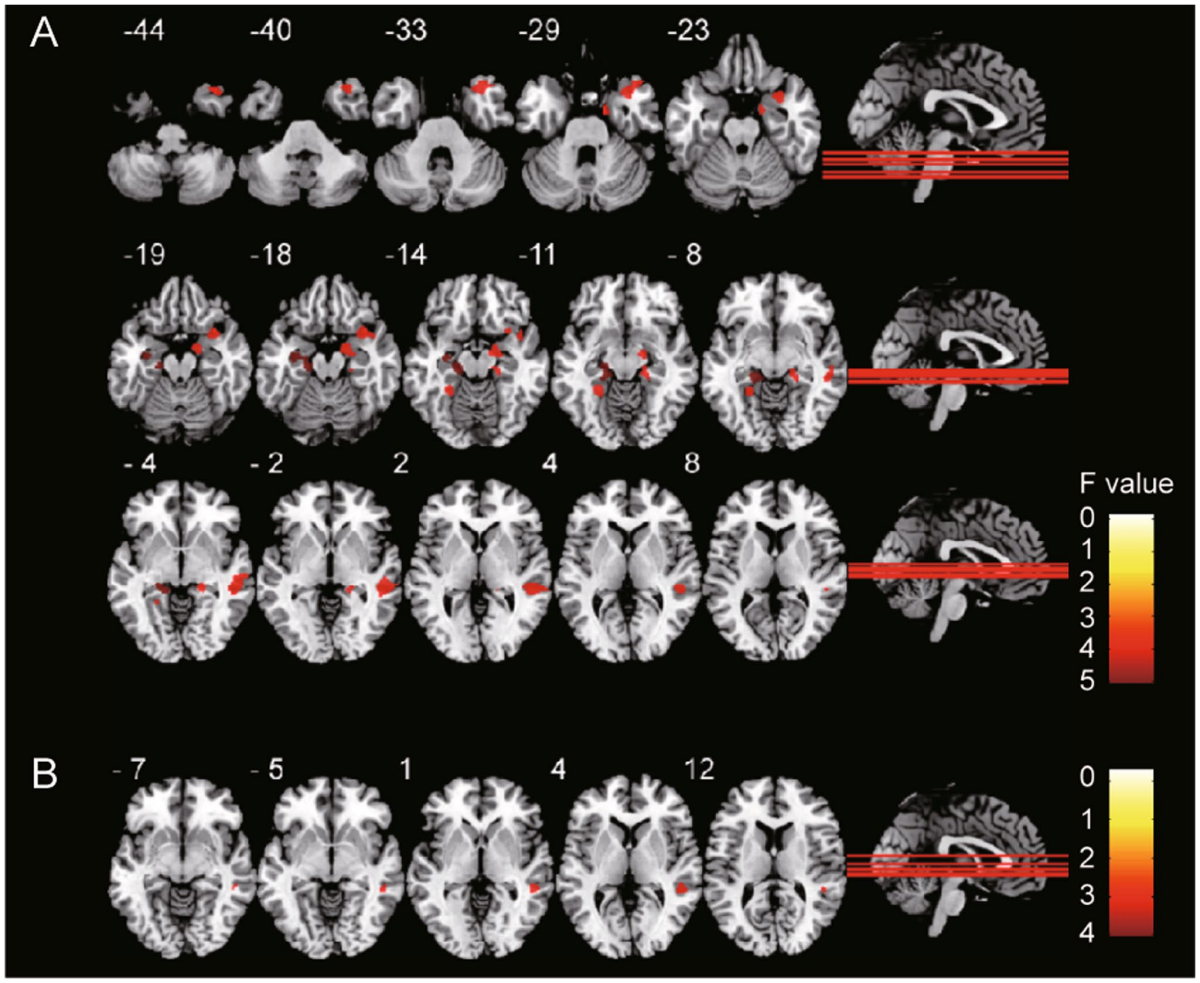
GMV differences among groups. The gray matter volume of **(A)** T2DM with HGL group and **(B)** T2DM with NGL group was significantly (cluster-level FDR correction, *p* < 0.05) decreased compared with that in the HC group. The color bar shows the range of *F* values.

Compared with the HC group, the T2DM with HGL group showed significantly decreased GMV in the bilateral parahippocampal gyrus (PHG), right middle temporal gyrus (MTG), parahippocampal gyrus (PHG), temporal pole (TPOmid), hippocampus (HIP), and left lingual gyrus. The T2DM with NGL group also showed decreased GMV in the right superior temporal gyrus (STG) compared with the HC group. No notable variations were discovered between the T2DM with HGL and T2DM with NGL groups in GMV (Table 2; Figure 1). When age, gender, and education level were used as covariates to recalculate the differences in brain areas among the three groups, we found that the significantly different brain regions were still concentrated in the superior temporal gyrus, middle temporal gyrus, hippocampus, and parahippocampal gyrus. The details are presented in Supplementary Table S1.

**Table 2:** Brain regions with reduced gray matter volume in the T2DM patients with HGL and T2DM patients with NHL compared with the healthy controls.

Condition	Brain regions	Cluster size	*F* score of the peak voxel	MNI coordinates of the peak voxel (mm)		
				*x*	*y*	*z*
HGL < HC	Right middle temporal gyrus	756	4.22	54	−31.5	−4.5
	Left parahippocampal gyrus	720	5.14	−15	−24	−15
	Left lingual gyrus	208	4.10	−22.5	−45	−9
	Right temporal pole	1,051	4.49	40.5	21	−34.5
	Right parahippocampal gyrus	284	4.31	21	−25.5	−16.5
	Right hippocampus	474	4.05	16.5	−7.5	−13.5
NGL<HC	Right superior temporal gyrus	362	4.30	48	−37.5	9

HGL, T2DM patients with HGL; NGL, T2DM patients with NGL; HC, healthy controls.

### 3.3. Correlation of gray matter volume with serum P-tau-181 in the diabetes mellitus group

**Figure 2:**
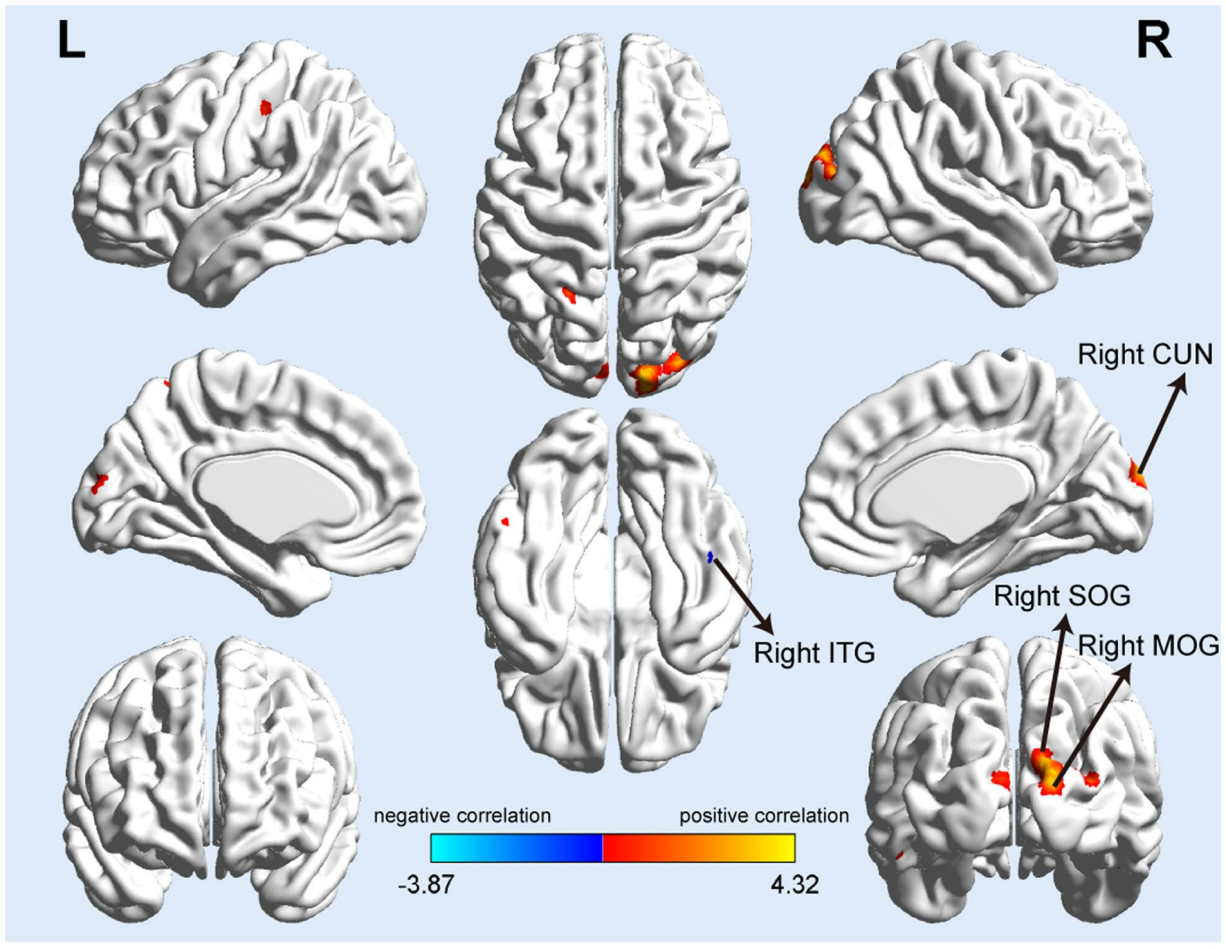
Serum P-tau-181 correlated with gray matter volume in T2DM patients. The mean GMV values of the right superior and middle occipital gyrus (SOG and MOG), right cuneus (CUN) showed significantly positive correlations with serum P-tau-181, and the GMV of the right inferior temporal gyrus (ITG) was significantly negatively correlated with serum P-tau-181.

Correlation analysis showed that GMV in many brain regions correlated with P-tau-181 levels in all T2DM patients. There were positive and negative correlations between GMV and serum P-tau-181 values in the T2DM groups (*p* < 0.001). The GMV of the right superior occipital gyrus (SOG), cuneus (CUN), and middle occipital gyrus (MOG) showed significantly positive correlations with serum P-tau-181, and the GMV of the right inferior temporal gyrus (ITG) was significantly negatively correlated with P-tau-181 (Table 3; Figure 2). We also investigated the combined effects of P-tau-181 and HbA1c on GMV using multivariate analysis, and the detailed results are shown in Supplementary Table S2.

**Table 3:** Brain regions significantly associated with P-Tau-181 in T2DM patients.

cerebral hemisphere	Brain regions	*T* score of the peak voxel	Cluster size	MNI coordinates of the peak voxel (mm)		
				*x*	*y*	*z*
Areas of positive correlation between P-Tau-181 and gray matter volume in patients with T2DM						
Right	Superior occipital gyrus	4.3184	173	16.5	−99	15
Right	Cuneus	4.3184	105	16.5	−99	15
Right	Middle occipital gyrus	3.8897	95	28.5	−88.5	16.5
Areas of negative correlation between P-Tau-181 and gray matter volume in patients with T2DM						
Right	Inferior temporal gyrus	3.8733	25	51	−33	−18

The voxel-wise correlation between GMV and serum P-tau-181 for all T2DM patients was analyzed using multiple regression (cluster-level FDR<0.05).

### 3.4. The results of ROC curve analysis

**Figure 3:**
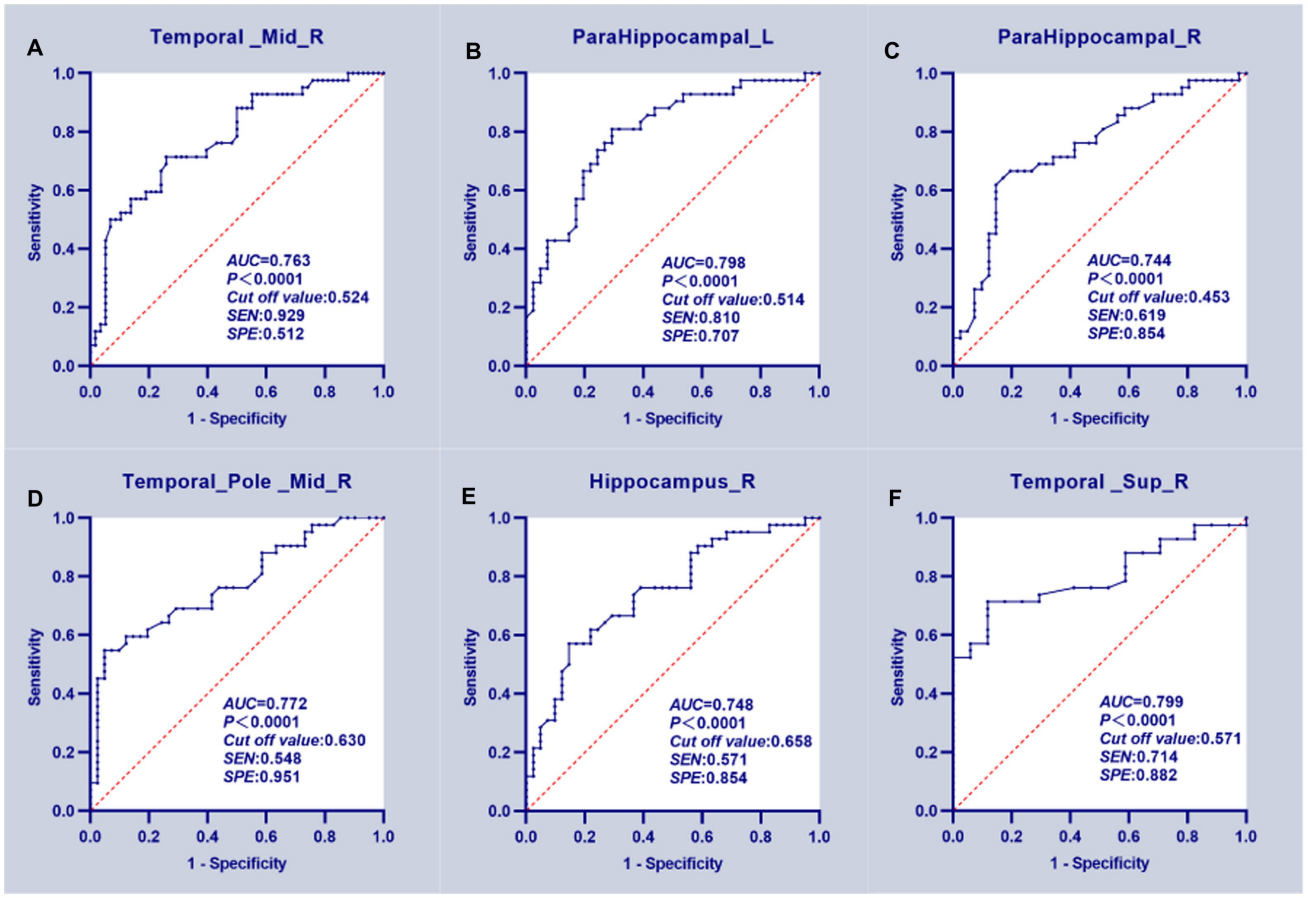
ROC curve analysis results. **(A–E)** The mean GMV values of five clusters which can significantly (*p* < 0.0001 for AUC value) distinguish T2DM patients with HGL from the HC group. **(F)** The GMV of the right superior temporal gyrus significantly discriminated T2DM patients in the NGL and HC groups.

According to the ROC curves, the GMV of the bilateral PHG, right MTG, TPOmid, and HIP can distinguish the T2DM with HGL group from the HC group; all attained a significance level of *p* < 0.001 for AUC. Using a single ROI, the DeLong test showed no significant differences in the AUC values, suggesting that they were equally effective at distinguishing T2DM with HGL and HC groups (*p* > 0.05). Furthermore, the GMV of the right STG could discriminate the T2DM with NGL and HC groups. The cutoff values of each value in the different ROIs were chosen according to the Youden index (Table 4; Figure 3).

**Table 4:** The statistics of ROC curve analysis for altered brain clusters that distinguish the T2DM patients with HGL from the T2DM patients with NGL and healthy controls.

Clusters	*P* value	Cutoff value	SEN	SPE	AUC	95% CI
HGL vs. HC						
Right middle temporal gyrus	<0.0001	0.524	0.929	0.512	0.763	0.683–0.868
Left parahippocampal gyrus	<0.0001	0.514	0.810	0.707	0.798	0.702–0.894
Right temporal pole	<0.0001	0.630	0.548	0.951	0.772	0.671–0.873
Right parahippocampal gyrus	<0.0001	0.453	0.619	0.854	0.744	0.636–0.851
Right hippocampus	<0.0001	0.658	0.571	0.854	0.748	0.643–0.853
NGL vs. HC						
Right superior temporal gyrus	<0.0001	0.571	0.714	0.882	0.799	0.688–0.910

HGL, T2DM patients with HGL; NGL, T2DM patients with NGL; HC, healthy controls; SEN, sensitivity; SPE, specificity; AUC, The area under the curve; 95% CI, 95% Confidence interval.

## 4. Discussion

In the present study, we investigated the differences in GMV between T2DM patients with different levels of HbA1c and healthy controls and further analyzed the correlation between brain GMV and serum P-tau-181 levels in all T2DM patients. The results showed that regardless of how high or low the level of HbA1c was in patients with T2DM, the brain GMV was decreased, and the increase in serum P-tau-181 was significantly correlated with the change in GMV, which suggested that regardless of glycemic control, T2DM patients should be treated as soon as possible to minimize brain damage. In addition, serum P-tau-181 concentrations are increased in T2DM patients, and more studies are needed to clarify the role of serum P-tau-181 as a potential biomarker of diabetic mild cognitive impairment.

There was no significant difference in GMV between the T2DM with HGL and NGL groups, which is similar to a previous study of functional MRI parameters in T2DM with HGL and NGL groups that showed no significant difference in regional homogeneity (ReHo) and amplitude of low-frequency fluctuation (ALFF) indicators between the two groups, indicating that there was no significant difference in the activity level of neurons in brain tissue between the two groups (Zhang et al., 2021). Since most of the GM is made up of the soma and dendrites of neurons, it is reasonable to assume that the structure of the GM in patients with high and normal HbA1c levels is similar. However, GMV was significantly decreased in both T2DM groups compared with the HC group. We further found that the brain GMV of the T2DM with HGL group decreased mainly in the right MTG, PHG, right TPOmid, and right hippocampus; the GMV of right the STG in the T2DM with NGL group was decreased. This is consistent with previous studies showing a decrease in GMV in DM patients. In previous cross-sectional investigations, right temporal cortex and hippocampus volumes in individuals with T2DM have been found to be decreased (Chen et al., 2012; Zhang et al., 2014). Since hyperglycemia may produce a variety of metabolic and molecular changes, including brain cell malfunction or death, it is thought to be the main cause of diabetes-related disturbances in the brain. Consequently, the ensuing neuronal death may be detected on MRI as extensive atrophy (Tomlinson and Gardiner, 2008; Liu et al., 2017; Yao et al., 2021; Cui et al., 2022). The default mode network (DMN), which includes the MTG, has been strongly associated with attention, working memory, language processing, voice production, and related processes (Fox et al., 2006). The shift from normal cognition to moderate cognitive impairment in T2DM patients is accelerated by MTG atrophy, which is linked to the neurodegeneration of the disease and cognitive dysfunction (Zhang et al., 2014; Wu et al., 2017).

The hippocampus is a growth of the cerebral cortex’s edge and is in charge of memory and learning (Squire, 1992). The hippocampus is extremely vulnerable to hyperglycemia (Sonneville et al., 2012; Yun et al., 2021) with direct neurotoxic consequences, such as increased polyol pathway flow, oxidative stress, and elevated development of advanced glycation end products (AGEs), all of which have been linked to neuronal disease (Gispen and Biessels, 2000). Furthermore, spatial memory and cognition are impaired in diabetic animal models, and hippocampal neuronal death is also promoted (Sadeghi et al., 2016). The development of memories for highly processed sensory information may be greatly influenced by the parahippocampal area in general (Engelien et al., 2000). The PHG is one of the main hubs of the DMN in the medial temporal lobe during the resting state according to previous studies, and injury to the PHG has been proven to cause considerable memory problems (Suzuki et al., 1993; Ward et al., 2014). Past studies have shown that in diabetics, reduced insulin levels lead to a disorder in insulin signaling, excessive deposition of hyperphosphorylated tau, and ultimately atrophy of the hippocampus and parahippocampal region, resulting in a series of clinical symptoms (Adachi et al., 2021). In fact, hippocampal damage occurs throughout the course from impaired fasting glucose levels to diabetes (Convit et al., 2003; Lamport et al., 2009). Long-term hyperglycemia can result in a series of metabolic changes in the body, such as the accumulation of AGEs and the creation of reactive oxygen species, as well as oxidative stress and the production of various inflammatory factors. It can also damage brain neurons and eventually cause gray matter atrophy (Chen et al., 2017). On the other hand, people with T2DM who have persistently high HbA1c levels are more likely to develop vascular problems such retinopathy, atherosclerosis, peripheral artery disease, and cerebrovascular disease. Hypoperfusion of nearby brain tissues will result from damage to cerebral microvessels (Paneni et al., 2013). In addition, HbA1c levels are consistent with blood glucose levels and diabetes-related complications (Xu et al., 2014). Therefore, the damage to cerebrovascular integrity may be the underlying mechanism of brain atrophy in T2DM patients. In addition, although there was no significant GMV difference between the two T2DM groups, the two groups of diabetic patients showed different volume atrophy of brain regions compared with the healthy group, suggesting that diabetic brain atrophy is specific to hyperglycemia. In a study of factors associated with brain atrophy in normal elderly subjects, HbA1c was identified as a risk factor for a higher incidence of brain atrophy (Enzinger et al., 2005). This confirms our finding that patients in the hyperglycemia group are characterized by more brain atrophy. Additionally, ROC curve research showed that the GMVs of the bilateral PHG, right MTG, TPOmid, HIP, and STG, which may be useful as diagnostic biomarkers, had excellent classification performance for separating T2DM patients from HC subjects.

The deposition of amyloid-protein (Aβ) in the brain parenchyma and phosphorylated tau deposition in NFTs in cerebral neurons are two important features of Alzheimer’s disease (AD) (Zou et al., 2020). Tau protein is essential to maintain the stability of microtubules in neurons of the central nervous system (Khan and Bloom, 2016). Instead, hyperphosphorylated tau dissociates and forms insoluble aggregates in neurons called NFTs, leading to loss of synapses and impaired neuronal function, which together with Aβ drive neurodegeneration (Daly et al., 2000; Calvo-Flores Guzman et al., 2020). It has been established that diabetes and AD have a number of shared pathogenic factors (Jash et al., 2020; Sun et al., 2020). These processes might encourage Aβ and phosphorylated tau protein to play an important role in the development of diabetes. An increasing number of studies have shown that the longitudinal increase in serum P-tau-181 is significantly associated with abnormal cerebrospinal fluid biomarker levels, structural imaging atrophy and cognitive deterioration (Nam et al., 2020; Zou et al., 2020; Chen et al., 2021). It has been further demonstrated that plasma P-tau-181 can be used to detect cerebral amyloidosis not only in clinical AD but also in asymptomatic AD patients (De Meyer et al., 2022). Therefore, we collected the serum P-tau-181 of healthy subjects and T2DM patients and tried to reveal the relationship between serum P-tau-181 and diabetic brain damage.

Some studies have found that atrophy of the occipital lobe in T2DM patients is mainly located in the bilateral lateral occipital lobe, lingual gyrus and precuneus (Chen et al., 2017). The lingual gyrus and the middle occipital gyrus are considered to be the brain regions related to the processing of visual information and the encoding of visual memory (Cui et al., 2016). The inferior temporal gyrus also contributes significantly to visual perception (Buckley et al., 1997). These abnormalities in the occipital and inferior temporal lobes may be due to underlying visual impairment, a well-known complication associated with T2DM and diabetic retinopathy. We further found that serum P-tau-181 was positively correlated with the GMV of the right SOG, MOG and CUN but negatively correlated with the GMV of the right ITG for all T2DM patients. In a longitudinal study of Parkinson’s disease (PD) (the average age was 60.89 years), a transient decrease in CSF P-tau was observed in the early stages of the disease (Dolatshahi et al., 2018). It is speculated that this is due to the toxic P-tau in neurons in the early stage of the disease in the form of NFTs, which leads to the compensatory absorption of more functional tau molecules by cells to reverse the function of neuronal transportation; this hypothesis has been verified regarding the ot-syn protein in PD (Førland et al., 2018). In addition, a study on AD demonstrated that serum P-tau-181 was significantly positively correlated with CSF P-tau-181, suggesting that P-tau-181 in plasma should be of brain origin (Tatebe et al., 2017). As a disease with NFTs, the transient decrease in CSF P-tau in PD patients of the same age group may also occur in T2DM patients of the same age group, and the serum P-tau-181 content in T2DM patients may also decrease transiently. If this phenomenon is finally confirmed, the positive correlation between GMV and serum P-tau-181 levels in T2DM patients in this study may also be explained. In addition, a study of nonlinear changes in GMV in AD patients suggests that increased GMV in some brain regions may be due to early neuroinflammation associated with amyloid accumulation, followed by recovery at the onset of neurodegenerative processes associated with tau accumulation (Gispert et al., 2015). DM, which demonstrates the same amyloid accumulation as AD, may also exhibit an increase in GMV associated with neuroinflammation in the early stage of the disease, and when P-tau-181 accumulates excessively, GMV in some brain regions begins to decrease. This also supports the correlation between GMV and serum P-tau levels in DM patients proposed in this study.

The main limitation of this study is that it is a cross-sectional study with a relatively small sample size. In the future, a longitudinal study will be needed to evaluate the relationship between diabetic regional brain atrophy and relevant clinical variables in a larger sample. In addition, due to the pattern of participation, the patients in this study were relatively young, and we could not reject the possibility that the results might only appear in young T2DM patients; at the same time, the imaging and pathological changes must be noted as being possibly characteristic of young patients with T2DM. This study also found bilateral cerebral hemisphere asymmetry in the changes in GM structure in T2DM patients. In the future, we intend to complete our study by performing specific tests on subjects in multiple cognitive domains to analyze a specific cognitive function and further study the white matter structures or the functional metabolic changes related to this asymmetry to better explain correlations with the clinical characteristics of T2DM patients.

## 5. Conclusion

In summary, we confirmed the differences in GMV between T2DM patients with different HbA1c level and healthy subjects, and suggested that GMV atrophy can be used as a potential biological indicator of T2DM. In addition, we found a significant correlation between serum P-tau-181 and GMV in T2DM patients, indicating the critical role that P-tau-181 plays in diabetic encephalopathy and providing new insights into the neuropathological mechanism of diabetic encephalopathy. This study demonstrates that patients with T2DM, regardless of HbA1c level, should receive active and reasonable intervention as soon as possible to reduce or block further T2DM-related deterioration.

## Data availability statement

The original contributions presented in the study are included in the article/Supplementary materials, further inquiries can be directed to the corresponding author.

## Ethics statement

The studies involving human participants were reviewed and approved by the Shandong Provincial Hospital Affiliated to Shandong First Medical University Subcommittee on Human Studies Institutional Review Board examined and approved the study.

## Author contributions

YG wrote the main manuscript text. CS, BC, HX, YC, XZ, NW, and YW prepared the clinical data and imaging data. CL revised the main manuscript text. All authors contributed to the article and approved the submitted version.

## Funding

This work was supported by grants from the Shandong Provincial Natural Science Foundation (ZR2020MH288).

## Conflict of interest

The authors declare that the research was conducted in the absence of any commercial or financial relationships that could be construed as a potential conflict of interest.

## Publisher’s note

All claims expressed in this article are solely those of the authors and do not necessarily represent those of their affiliated organizations, or those of the publisher, the editors and the reviewers. Any product that may be evaluated in this article, or claim that may be made by its manufacturer, is not guaranteed or endorsed by the publisher.

## Footnotes

^1^ www.mocatest.org

^2^ http://www.fil.ion.ucl.ac.uk/spm

^3^ http://www.alivelearn.net/xjview

^4^ https://www.medcalc.org
