# Supplementary material for: Voxel-based morphometry reveals the correlation between gray matter volume and serum P-tau-181 in type 2 diabetes mellitus patients with different HbA1c levels
Source: Front Neurosci. 2023 May 15;17:1202374. doi: 10.3389/fnins.2023.1202374 (PMC10225590; doi:10.3389/fnins.2023.1202374)
Supplement: Supplementary file 1 [file Data_Sheet_1.docx]

**Supplementary test results**

**2. Materials and Methods**

**2.1. Subjects**

The inclusion criteria for all patients were as follows: (1) an age range from 40 to 70 years; (2) junior high school education or higher; and (3) right-handedness. Patients were excluded from the study if they had (1) left-handedness; (2) neuropathies caused by other reasons; (3) brain trauma, surgery or brain tumors; (4) acute complications of T2DM and severe hypertension; (5) history of any serious cerebrovascular, neurological or psychiatric diseases; (6) abuse of alcoholism or drugs; or (7) any contraindications to MRI. The inclusion criteria for the HCs were as follows: (1) age 40-70 years, with junior high school education or above; (2) right-handed; (3) no history of diabetes and glycated hemoglobin (HbA1c) level of between 4 and 6%; (4) no history of any serious medical, psychiatric or neurologic diseases; (5) no history of head trauma, surgery, tumors or loss of consciousness; and (6) absence of alcohol or drug abuse.

**2.5.** **Statistical analysis**

**Analysis of the differences in GMV among groups:** To analyze the differences in GMV images among the three groups, voxel-wise one-way analysis of covariance (ANCOVA) was performed using SPM 8, with age, gender and education level as covariates, and the analysis was constrained in binary AAL template as mask.

**Multivariate analysis of gray matter volume in diabetic patients:** Multivariate analysis of whole-brain voxel-level was performed using SPM 8 with P-tau-181 and HbA1c as independent variables, GMV of all diabetic patients as dependent variable, age and gender as covariates, and binary AAL template as mask.

**3. Results**

**3.2** **Altered gray matter volume among groups**

When further adding education level as a covariate in ANCOVA, the significant results were derived after using the cluster-level familywise error (FWE) multiple comparisons correction (cluster-wise threshold of q<0.05 based on an uncorrected voxel-wise threshold of p<0.005). We found that the brain regions were concentrated in the superior temporal gyrus, middle temporal gyrus, hippocampus and parahippocampal gyrus. The details are presented in **Supplementary Table S1**.

**3.4. Correlation of gray matter volume with serum P-tau-181 in the diabetes mellitus group**

When P-tau-181 and HbA1c were taken into account for their combined effects on GMV, we discovered that the right superior occipital gyrus and right middle occipital gyrus had positive correlations with both Tau-p-181 and HbA1c, whereas the right inferior temporal gyrus had negative correlations. However, no significant clusters remained after cluster-level FDR correction (voxel-wise p<0.001, cluster-wise q<0.05). The details are presented in **Supplementary Table S2**.

**Supplementary Table S1.** **Significant differences in GMV among groups.**

|  | | Brain  regions | Cluster  size | | z score of the  peak voxel | | MNI coordinates of the peak voxel | | | | | | | |
| --- | --- | --- | --- | --- | --- | --- | --- | --- | --- | --- | --- | --- | --- | --- |
|  |  |  |  |  |  |  | x | | y | | z | | | |
|  | Right superior temporal gyrus | | | 480 | | 4.17 | | 40.5 | | 9 | | -45 |  |  |
|  | Right middle temporal gyrus | | | 282 | | 4.17 | | 40.5 | | 9 | | -45 | |  |
|  | Right parahippocampal gyrus | | | 19 | | 4.17 | | 40.5 | | 9 | | -45 | |  |
|  | Left hippocampus | | | 702 | | 3.77 | | -19.5 | | -21 | | -22.5 | |  |
|  | Left parahippocampal gyrus | | | 300 | | 3.77 | | -19.5 | | -21 | | -22.5 | |  |
|  | Right middle temporal gyrus | | | 862 | | 3.77 | | 43.5 | | -10.5 | | -16.5 | |  |
|  | Right superior temporal gyrus | | | 616 | | 3.77 | | 43.5 | | -10.5 | | -16.5 | |  |

**Notes:** The significant results were derived after using the cluster-level familywise error (FWE) multiple comparisons correction (cluster-wise threshold of q<0.05 based on an uncorrected voxel-wise threshold of p<0.005).

**Supplementary Table S2.** **Brain regions significantly associated with P-Tau-181 and HbA1c in T2DM patients**

| cerebral hemisphere | Brain regions | z score of the  peak voxel | Cluster  size | | | MNI coordinates of the peak voxel | | | |
| --- | --- | --- | --- | --- | --- | --- | --- | --- | --- |
|  |  |  |  | | x | | y | z |  |
| Areas of positive correlation between P-Tau-181, HbA1c and gray matter volume in patients with T2DM | | | | | | | | |  |
| Right | Superior occipital gyrus | 3.98 |  | 199 | 18 | | -103.5 | 12 |  |
| Right | Cuneus | 3.98 |  | 83 | 18 | | -103.5 | 12 |  |
| Areas of negative correlation between P-Tau-181, HbA1c and gray matter volume in patients with T2DM | | | | | | | | |  |
| Right | Inferior temporal gyrus | 3.58 |  | 26 | 51 | | -36 | -21 |  |

**Notes:** Multivariate analysis between GMV, HbA1c and serum P-tau-181 for all T2DM patients (the current results are based on no correction).
